# Thresholds for clinical importance for four key domains of the EORTC QLQ-C30: physical functioning, emotional functioning, fatigue and pain

**DOI:** 10.1186/s12955-016-0489-4

**Published:** 2016-06-07

**Authors:** Johannes M. Giesinger, Wilma Kuijpers, Teresa Young, Krzysztof A. Tomaszewski, Elizabeth Friend, August Zabernigg, Bernhard Holzner, Neil K. Aaronson

**Affiliations:** Division of Psychosocial Research & Epidemiology, The Netherlands Cancer Institute, Plesmanlaan 121, 1066 CX Amsterdam, The Netherlands; Lynda Jackson Macmillan Centre, Mount Vernon Cancer Centre, Rickmansworth Rd, Northwood, HA62RN UK; Department of Anatomy, Jagiellonian University Medical College, Krakow, Poland; Hepatobiliary Research, The Ark Centre, Basingstoke & North Hampshire Hospital, Aldermaston Road, Basingstoke, RG24 9NA UK; Department of Internal Medicine, Kufstein County Hospital, Endach 27, A-6330 Kufstein, Austria; Department of Psychiatry and Psychotherapy, Innsbruck Medical University, Anichstr. 35, A-6020 Innsbruck, Austria

**Keywords:** Threshold for clinical importance, EORTC QLQ-C30, Patient-reported outcomes, Quality of life, Cancer

## Abstract

**Background:**

The EORTC QLQ-C30 is one of the most widely used quality of life questionnaires in cancer research. Availability of thresholds for clinical importance for the individual questionnaire domains could help to increase its interpretability. The aim of our study was to identify thresholds for clinical importance for four EORTC QLQ-C30 scales: Physical Functioning (PF), Emotional Functioning (EF), Pain (PA) and Fatigue (FA).

**Methods:**

We recruited adult cancer patients from Austria, the Netherlands, Poland and the UK. No restrictions were placed on diagnosis or type or stage of treatment. Patients completed the QLQ-C30 and three anchor items reflecting potential attributes of clinically important levels of PF, EF, PA and FA. We merged the anchor items assessing perceived burden, limitations in daily activities and need for help into a dichotomous external criterion to estimate thresholds for clinical importance using Receiver Operator Characteristic (ROC) analysis.

**Results:**

In our sample of 548 cancer patients (mean age 60.6 years; 54 % female), the QLQ-C30 scales showed high diagnostic accuracy in identifying patients reporting burden, limitations and/or need for help related to PF, EF, PA and FA. All areas under the curve were above 0.86.

**Conclusions:**

We were able to estimate thresholds for clinical importance for four QLQ-C30 scales. When used in daily clinical practice, these thresholds can help to identify patients with clinically important problems requiring further exploration and possibly intervention by health care professionals.

## Background

For the evaluation of cancer treatments, parameters such as survival time or time to tumor progression are the most common primary outcomes. In addition, assessment of patient-reported physical or psychosocial symptom burden has gained considerable importance within clinical trials [1–4] and clinical practice [5]. Patient-reported outcomes (PROs) can add important information to clinical trials and medical decision making [6]. To improve the interpretation of PRO data collected within clinical trials, research efforts have focused on thresholds like minimally important differences (MIDs) or minimally important changes (MICs) over time. An MID or MIC is the smallest difference or change in PRO score points considered to be of clinical relevance [7]. Such thresholds related to change scores help to interpret the impact of disease and treatment in a relative manner by comparison to previous assessments of the same patient or to other patient groups. However, they do not aid in the interpretation of absolute scores, i.e. scores obtained from an individual patient or a patient group at a single time point. To better understand what a given score from an individual patient or an average score from a patient group means, researchers have started to develop clinical thresholds that allow identification of clinically important symptom burden or impairment [8, 9].

The EORTC QLQ-C30 [10] is one of the most widely used health-related quality of life (HRQOL) questionnaires in cancer research. It assesses important functioning domains (e.g. physical, emotional, role) and common cancer symptoms (e.g. fatigue, pain, nausea/vomiting, appetite loss). Current approaches to defining clinical thresholds for the EORTC QLQ-C30 vary substantially and each has its own strengths and weaknesses. The simplest and most straight-forward approach is to rely on the wording of the item or response categories themselves, and classify a patient as having a clinically important problem if s/he responds with at least “a little” for any given item or domain assessed on the 4 point response scale (i.e. “not at all,” “a little,” “quite a bit,” and “very much”) [11, 12]. This approach can be problematic as it uses the same threshold for all QLQ-C30 domains. As has been shown in Item Response Theory analyses [13], the QLQ-C30 items differ in item difficulty (i.e. they assess different levels of severity of a problem or symptom), suggesting that scores are not directly comparable across domains. For example, reporting “quite a bit” of vomiting probably indicates a different symptom level than reporting “quite a bit” of trouble with sleeping. This problem also applies to studies using the same cut-off score as a threshold for all domains (e.g. a score of 50 as done by Klinkhammer et al. [14]).

Another approach is based on score distributions and makes use of percentiles or statistics from reference populations (often the general population). However, the choice of a percentile or statistic is somewhat arbitrary. In addition, if, as is commonly done [15–17], the general population mean is used as a threshold, the resulting percentage of patients with a clinically important problem is likely to be very high. Using the population average often means that around half of the general population (depending on the score distributions) will be classified as having a clinically important problem. Also, such a definition of clinical thresholds assumes the prevalence rate of clinically important problems is constant across the various HRQOL domains.

These limitations can potentially be overcome by relying on external anchors for determining thresholds for clinical importance. An example of this approach is the use of patients’ ratings of their most bothersome symptoms or functional limitations as an external anchor. Patients could be asked to name their most bothersome symptoms or limitations in addition to completing scales assessing symptom or problem levels. This allows one to determine thresholds above which patients are likely to consider a symptom or limitation as bothersome (e.g. using ROC analysis). Although this has clear advantages over distribution-based thresholds, it relies on a somewhat narrow definition of clinical importance (basically being equated with what is most “bothersome”). Also, in previous work the number of most bothersome symptoms per patient has been fixed *a priori* [18].

In the current study, we have investigated an alternative approach to establishing thresholds for clinical significance that does not rely on one, but rather on several potential anchors. The objective of our study was to use an anchor-based approach to defining thresholds for clinical importance for four key EORTC QLQ-C30 scales: Physical Functioning (PF), Emotional Functioning (EF), Pain (PA) and Fatigue (FA). Thresholds for clinical importance were intended to identify symptoms and functional health problems that require a health care professional’s attention.

## Methods

### Sample

We recruited a cross-sectional sample of cancer patients from centers in four European countries: the Netherlands Cancer Institute (the Netherlands), Kufstein County Hospital (Austria), the Mount Vernon Cancer Centre and Basingstoke & North Hampshire Hospital (the United Kingdom) and the Jagiellonian University Medical College (Poland). To obtain a heterogeneous sample we included any cancer patient (on- or off-treatment) aged above 18 years. Patients were invited to participate in the study via mail or during a clinic visit, and were asked to complete the EORTC QLQ-C30 and an additional questionnaire with anchor items.

### Assessment instruments

#### EORTC QLQ-C30

The EORTC QLQ-C30 [10] is an internationally validated and widely used cancer-specific HRQOL instrument. It contains five functioning scales (physical, social, role, cognitive, and emotional functioning), eight symptom scales (fatigue, nausea/vomiting, pain, dyspnea, sleep disturbances, appetite loss, constipation, and diarrhea), financial impact, and overall quality of life. All scale scores are linearly converted to range from 0 to 100. For the functioning scales and global QOL higher scores indicate better functioning; for the symptom scales higher scores indicate higher symptom burden.

#### Anchor items

An expert panel including four PRO researchers, three psycho-oncologists, three oncologists and a biostatistician generated a set of anchor items intended to assess the clinical importance of functional health problems and symptoms included in the QLQ-C30. This set of anchor items was reviewed independently by the EORTC Quality of Life Group as part of a grant review process and additionally underwent external anonymous peer review within this process. The anchor items were also presented and discussed in plenary at an EORTC Quality of Life Group meeting. The wording of the anchor items and the response categories were as follows:

- Has your PF/EF/FA/PA been a burden to you?
  - Not at all – A little – Quite a bit – Very much
- Has your PF/EF/FA/PA limited your daily activities?
  - Not at all – A little – Quite a bit – Very much
- Have you needed any help or care for your PF/EF/FA/PA?
  - No help – A little help from family or friends – Quite a bit of help from family or friends – Professional help (e.g. physicians, nurses)

A functional limitation or symptom was considered to be potentially clinically important if any of the three anchor items was answered positively. More specifically, a patient was considered to have a problem of at least “minimal clinical importance”, if s/he reported at least “a little” for any of the three anchor items. Patients who rated their problem/symptom as “quite a bit” or “very much” for any anchor item were classified as having a problem of “clinical importance”. In line with this, we labeled the two possible thresholds to be investigated further TMCI (Threshold for Minimal Clinical Importance) and TCI (Threshold for Clinical Importance).

### Statistical analysis

Sample characteristics are presented as means, standard deviations, and absolute and relative frequencies.

In a first step we calculated prevalence rates (i.e. relative frequency of positive cases) for symptoms and functioning problems in our sample based on the above definitions of TMCI and TCI. These prevalence rates were evaluated to decide which anchor definition (TMCI or TCI) to employ for further analysis and development of the final thresholds. We considered an anchor definition to be too sensitive if it resulted in the majority of patients being classified as positive cases on multiple domains. This would not be sustainable in clinical practice, as it would require additional help and/or intervention for too many patients.

To assess the discriminatory power of the QLQ-C30 scales, we calculated effect sizes (Cohen’s d) for the mean QLQ-C30 scale score differences between patients classified as having a clinically important problem and those not so classified. To determine the diagnostic accuracy of the QLQ-C30 scales with regard to the external anchors we conducted Receiver Operating Characteristic (ROC) analyses and calculated the Area Under the Curve (AUC). In line with Hosmer and Lemeshow (1989), we classified diagnostic accuracy as follows: <0.70 poor; 0.70–0.80 acceptable; >0.80 excellent [19].

Selection of threshold scores for the QLQ-C30 scales was based primarily on Youden’s J [20], i.e. the sum of sensitivity and specificity minus one. In case of different thresholds showing comparable Youden’s J, we chose the one providing higher sensitivity, as we considered sensitivity to be more important than specificity in the context of initial screening for problems at the individual patient level. We also calculated correlations (Spearman’s Rho) between the three anchor items.

We used binary logistic regression analysis to investigate invariance of diagnostic accuracy and stability of thresholds across different patient groups. The regression analysis included the dichotomous external anchor as the dependent variable, and the QLQ-C30 scale and the grouping variables (sex, age, stage, country, treatment status) as independent variables. In such a model, the main effect of the grouping variable indicates a difference in diagnostic accuracy of the anchor-based threshold (sensitivity/specificity) between the patient groups. A significant interaction term (grouping variable * scale) indicates that the optimal threshold providing the lowest misclassification rate differs across patient groups. To account for multiple testing in these sensitivity analyses we considered *p*-values below 0.01 to be statistically significant.

## Results

### Patient characteristics

Between October 2013 and September 2014 we recruited 548 patients (236 in the Netherlands, 151 in Austria, 100 in Poland and 61 in the UK). Mean age was 60.6 years (SD 12.3) and 54.0 % was female. The most frequent diagnoses were breast cancer (25.7 %), colorectal cancer (12.8 %) and lung cancer (11.7 %). Most patients had advanced disease (UICC stage III or IV: 61.2 %). At the time of the assessment 73.3 % of the patients were receiving anti-cancer treatment, with chemotherapy (24.7 %), chemotherapy and surgery (18.4 %), and chemo- and radiotherapy (11.4 %) being the most common treatments. Further details are given in Table 1.

**Table 1:** Descriptive statistics for sociodemographic and clinical variables (*n* = 548)

Age:	Mean (SD)	60.6 (12.3)
	Range	19–89
Sex:	Women	54.0 %
	Men	46.0 %
Diagnosis:	Breast cancer	25.7 %
	Colorectal cancer	12.8 %
	Lung cancer	11.7 %
	Head & neck cancer	8.0 %
	Prostate cancer	7.4 %
	Non-Hodgkin Lymphoma	7.3 %
	Stomach/Oesophagic cancer	6.6 %
	Gynaecologic cancer	6.0 %
	Other	14.5 %
UICC stage:	I	9.9 %
	II	28.9 %
	III	24.4 %
	IV	36.8 %
Current treatment:	No treatment	26.7 %
	Chemotherapy	24.7 %
	Chemotherapy and surgery	18.4 %
	Radio- and chemotherapy	11.4 %
	Surgery and Radiotherapy	4.4 %
	Surgery	4.0 %
	Other	10.4 %

### Anchor items and clinical importance

Burden, limitations or need for help were found to be most prevalent for PF and FA. The highest percentages of patients selecting one of the two most severe categories on the anchor items were found for limitations regarding FA (34.4 % of the patients answered “quite a bit” or very much”) and burden related to PF (33.9 % of the patients answered “quite a bit” or “very much”). Please see Table 2 for further details. Correlations between the three anchor items were generally high. The domain-specific correlations between reported burden and limitations in daily activities ranged from 0.75 for emotional functioning to 0.88 for pain. Correlations with need for help were lower ranging from 0.56 to 0.65 for burden, and from 0.61 to 0.72 for limitations (Table 3).

**Table 2:** Descriptive statistics for the QLQ-C30 scales and the anchor items (*n* = 548)

	Physical Functioning	Emotional Functioning	Fatigue	Pain
Mean (SD)	73.9 (23.8)	73.0 (24.5)	41.8 (27.6)	24.3 (28.8)
Burden:				
not at all	29.2 %	43.4 %	27.3 %	57.2 %
a little	37.0 %	35.2 %	40.5 %	25.1 %
quite a bit	24.8 %	14.2 %	23.0 %	13.5 %
very much	9.1 %	7.2 %	9.2 %	4.2 %
Limitation:				
not at all	28.8 %	57.6 %	29.1 %	59.0 %
a little	38.1 %	25.5 %	36.5 %	23.1 %
quite a bit	22.0 %	11.3 %	26.3 %	13.9 %
very much	11.1 %	5.7 %	8.1 %	4.1 %
Need for help:				
no help	57.1 %	66.9 %	59.7 %	71.4 %
little help (family)	21.8 %	17.0 %	23.3 %	13.1 %
quite a bit of help (family)	14.8 %	8.3 %	12.6 %	5.9 %
professional help	6.3 %	7.8 %	4.4 %	9.6 %
Prevalence TMCI	77.7 %	61.1 %	77.3 %	47.5 %
Prevalence TCI	41.7 %	28.0 %	39.2 %	24.1 %
Effect size TCI^a^	1.56	1.69	1.80	2.33

^a^Effect size given as Cohen’s d for comparing negative and positive cases as defined by the criteria for moderate/severe problems

**Table 3:** Correlations between anchor items (Spearman’s Rho)

	Physical Functioning	Emotional Functioning	Fatigue	Pain
Burden – limitations	0.83	0.75	0.83	0.88
Burden – help	0.56	0.62	0.59	0.65
Limitations – help	0.61	0.64	0.63	0.72

All *p* < 0.001

Using our definition for TMCI, we found a prevalence rate in our sample of 77.7 % for PF, 77.3 % for FA, 61.1 % for EF, and 47.5 % for pain. As expected, using the TCI definition, reflecting a higher degree of burden, limitations and need for help, we found lower prevalence rates: 41.7 % for PF, 39.2 % for FA, 28.0 % for EF, and 24.1 % for PA.

Effect sizes for mean differences between cases and non-cases based on the TCI classification ranged from 1.56 for PF to 2.33 for PA. Further details are given in Table 2.

The very high prevalence rates derived from the TMCI definition indicated that those thresholds are probably too liberal to derive meaningful prevalence rates or to be useful for screening in daily clinical practice. Further analyses were therefore conducted for the TCI only.

### Thresholds for the QOL scales

**Fig. 1:**
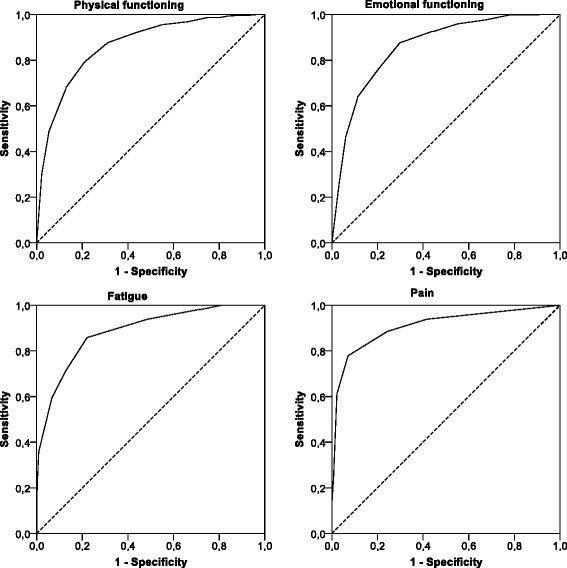
Receiver Operator Characteristic curves for the threshold for clinical importance

In the ROC analysis, the four QLQ-C30 scales showed high diagnostic accuracy in predicting the TCI criterion. AUC ranged from 0.86 for EF to 0.91 for PA, suggesting excellent screening properties [19] (Fig. 1).

Based on the rationale of selecting thresholds with high diagnostic accuracy in terms of Youden’s J and an emphasis on sensitivity, we obtained the following cut-off scores: PF = 83 points (sensitivity 0.87 and specificity 0.68), EF = 70 points (sensitivity 0.80 and specificity 0.76), FA = 39 points (sensitivity 0.86 and specificity 0.78), and PA = 25 points (sensitivity 0.89 and specificity 0.76). Please note that due to different scale directions clinically important scores for PF and EF are below the thresholds whereas for FA and PA they are above the thresholds. Further details are given in Table 4.

**Table 4:** Results from the Receiver Operator Characteristics (ROC) analysis and TCI

	Area under curve	95 % confidence interval	Threshold for Clinical Importance (TCI)	Sensitivity/Specificity
Physical Functioning	0.87	(0.83–0.90)	83	(0.87/0.68)
Emotional Functioning	0.86	(0.82–0.90)	70	(0.80/0.76)
Fatigue	0.89	(0.86–0.91)	39	(0.86/0.78)
Pain	0.91	(0.88–0.94)	25	(0.89/0.76)

### Sensitivity analysis

We did not find a significant interaction between the grouping variables (age, sex, tumour stage, treatment status, and country) and the QLQ-C30 scores when predicting caseness, which was defined according to the criteria for TCI. This suggests that diagnostic accuracy (in terms of AUC) is stable across patient groups.

The main effects of the grouping variables age, sex, tumor stage and treatment status were not statistically significant. Analysis of the impact of country showed that Poland differed significantly from the other countries with regard to EF (*p* = 0.005) and FA (*p* = 0.007). For EF, a cut-off of 62 points provided the highest value for Youden’s J for Poland and the other countries, but sensitivity and specificity was 0.80 and 0.81 for Poland and 0.64 and 0.89 for the other countries. Results were similar for FA, with the same cut-off of 39 points showing highest Youden’s J for both groups whereas the exact sensitivities and specificities differed (0.83 and 0.72 for Poland and 0.87 and 0.79 for the other countries).

## Discussion

In this study we have developed clinical thresholds for the physical functioning, emotional functioning, fatigue and pain scales of the EORTC QLQ-C30. By using three anchor items assessing burden, limitations and need for help related with a specific domain, we defined an external criterion for clinical importance. Applying this criterion in our sample, we found prevalence rates for clinically important problems ranging from 22.9 % (pain) to 38.5 % (physical functioning).

To investigate the invariance of diagnostic accuracy across different patient groups we conducted binary logistic regression analyses. These showed that differences across the investigated patient subgroups were minor, indicating invariance of the thresholds across these groups. Based on these findings we do not recommend using different thresholds for different subgroups (e.g. men versus women, patients on- and off-treatment). The invariance of thresholds is an important finding because the use of different thresholds for different patient subgroups would be complex in daily clinical practice and might not always be intuitively understood by health care professionals.

In principle, the anchor items used in our study could be used directly for screening for clinically important problems. However, using the QLQ-C30 and the developed thresholds instead provides additional benefits. The QLQ-C30 provides better measurement precision, in particular with regard to changes over time. It also allows one to relate individual patient scores to norm/reference data available from the literature and to use the collected data for scientific purposes. For these reasons clinicians and researchers may prefer using thresholds for this well-validated questionnaire over only using the anchor items from this study.

The methodological approach most comparable to our study is the analysis by Snyder et al. [8]. They established cut-off scores for the QLQ-C30 using unmet needs defined by dichotomised scores from the Supportive Care Needs Survey (SCNS) as the external criterion. In their study, the diagnostic accuracy of the six investigated QLQ-C30 scales (PF, EF, FA, PA, role functioning and global QOL) was found to be lower than in our study (AUCs were between 0.56 and 0.81). More interestingly, their thresholds were also substantially different from ours, with the differences ranging from 10 to 20 points. In their study, a lower symptom or functional health score was required to qualify as a positive case. This might be related to the use of a different anchor definition that was more sensitive than our TCI criterion. A patient was considered a positive case if s/he had a low, moderate or high need in a certain area, and a negative case if s/he had a satisfied need or if the question was not applicable. In our analysis, a more sensitive criterion like the TMCI would have led to prevalence rates of up to 78 %. In addition to this difference in the definition of the anchor items, the differences observed in optimal thresholds in their study and ours might indicate cross-cultural influences (American versus European cancer patients).

In another study by Snyder and colleagues [18], the QLQ-C30 was also shown to be capable of accurately identifying a patient’s most bothersome issues. For the prediction of these most bothersome issues, sensitivity and specificity for functional health were 0.75 and 0.64, respectively while for symptoms classification accuracy was somewhat better (sensitivity = 0.83; specificity = 0.76). Interestingly, the authors also found that diagnostic performance of change scores was lower than that for absolute scores. This is in line with a recent analysis of Snyder et al. [9] that showed that a change in patients’ QLQ-C30 scores does not relate consistently to a change in patients’ supportive care needs. Such findings support our approach relying on absolute scores rather than on change scores for identification of clinically important problems.

A limitation of our study is that the anchor items and the definitions for (minimal) clinical importance were based on input from an expert panel only. However, the three anchors that we employed in our study reflect characteristics of patients’ symptom burden and functional health limitations that are typically assessed in patient-reported outcome measures, either alone or in combination. Nevertheless, in future research it would be useful to elicit direct patient input on this issues. An additional potential limitation of the study is that the QLQ-C30 pain scale not only assesses severity, but also interference with daily activities. The larger area under the curve observed for the pain scale in comparison to the other three scales that we evaluated probably reflects, at least in part, this overlap of the pain scale with 2 of the 3 anchor items; the other three QLQ-C30 domains assess severity only and had overlap with only one anchor item.

A strength of our study is the large cross-cultural patient sample. In addition, we employed a rather broad definition of what makes a symptom or problem clinically important. We are aware that the set of anchor items is not exhaustive; however, in our study we found the three anchor items to be highly correlated (especially burden and limitations) and it is likely that additional anchor items (e.g. self-rated importance of a symptom) would be highly correlated with limitations, burden and need for help as well. We expect our thresholds to be fairly robust against changes of the criterion as long as correlations with our current anchor items are high.

## Conclusion

We believe that the thresholds for clinical importance developed for the four QLQ-C30 scales PF, EF, FA and PA will facilitate interpretation of these QOL scores on an individual- and on a group-level. The thresholds can be used to convert metric QOL scores to symptom prevalence rates in a meaningful way and thus allow comparison with prevalence rates collected with other methods such as clinicians’ ratings. In daily clinical practice such thresholds can guide identification of patients with clinically important problems that should be discussed with the patient. In a next step we will develop thresholds for clinical importance for all QLQ-C30 scales and the corresponding computer-adaptive measures currently being developed by the EORTC Quality of Life Group [21].

## Abbreviations

AUC, area under curve; EF, emotional functioning; EORTC, European Organisation for Research and Treatment of Cancer; FA, fatigue; HRQOL, health-related quality of life; MIC, minimal important change; MID, minimal important difference; PA, pain; PRO, patient-reported outcome; PF, physical functioning; QOL, quality of life; QLQ-C30, quality of life questionnaire - core 30; ROC, receiver operating characteristic; SD, standard deviation; SNCS, supportive care needs survey; TCI, threshold for clinical importance; TMCI, threshold for minimal clinical importance; UICC, union for international cancer control; UK, United Kingdom
